# The efficacy and safety of rituximab with or without glucocorticoid in inducing remission of MCD with different clinical presentations in adults: a retrospective study

**DOI:** 10.1093/ckj/sfae139

**Published:** 2024-05-03

**Authors:** Yujiao Sun, Zhuo Li, Jing Sun, Shasha Zhang, Rong Wang, Bing Chen

**Affiliations:** Department of Nephrology, Shandong Provincial Hospital Affiliated to Shandong First Medical University, Jinan, Shandong, China; Department of Nephrology, Shandong Provincial Hospital Affiliated to Shandong First Medical University, Jinan, Shandong, China; Department of Nephrology, Shandong Provincial Hospital Affiliated to Shandong First Medical University, Jinan, Shandong, China; Department of Nephrology, Shandong Provincial Hospital Affiliated to Shandong First Medical University, Jinan, Shandong, China; Department of Nephrology, Shandong Provincial Hospital Affiliated to Shandong First Medical University, Jinan, Shandong, China; Department of Nephrology, Shandong Provincial Hospital Affiliated to Shandong First Medical University, Jinan, Shandong, China

**Keywords:** glucocorticoid, minimal change disease, nephrotic syndrome, remission, rituximab

## Abstract

**Background:**

To investigate the efficacy and safety of rituximab (RTX) with or without glucocorticoid (GC) in inducing remission of minimal change disease (MCD) in adults.

**Methods:**

Twenty-one adult MCD patients were included in the study. The patients were assigned to the following three groups according to their background before RTX treatment: an RTX single drug direct induction treatment group (Group A; *n* = 9), a short-term, low-dose GC combined with RTX induction treatment group (Group B; *n* = 4), and a short-term, adequate-dose GC-induced remission and RTX maintenance treatment group (Group C; *n* = 8). The primary endpoints were the time to induction of remission and the rate of clinical remission at 12 months.

**Results:**

All patients achieved clinical remission, with 19 (90.48%) achieving complete remission (CR), and the median remission time was 4 (2.5, 12) weeks. Eight (88.89%) patients in Group A achieved CR, and the median remission time was 3 (2.25, 14) weeks. In Group B, three (75.00%) patients achieved CR, with a median remission time of 4 (4, 10) weeks. In Group C, eight (100.00%) patients achieved CR, and the median remission time was 3.5 (2, 4) weeks.

**Conclusions:**

In MCD patients without acute kidney injury, adequate RTX alone or short-term combined treatment with low-dose GCs can effectively induce and maintain MCD remission. Adequate short-term GCs combined with RTX maintenance may be an effective alternative for MCD patients in context of acute kidney injury. There is a need to investigate different induction therapy regimens for the remission of MCD patients with different backgrounds.

KEY LEARNING POINTS **What was known:**

- In recent years, rituximab (RTX) has primarily been used in the area of relapse reduction in patients with minimal change disease (MCD) who are corticosteroid dependent or have frequent relapses. This report aimed to investigate the efficacy and safety of RTX with or without glucocorticoid (GC) in inducing remission of MCD in adults.

**This study adds:**

- This study confirmed that adequate RTX alone or short-term combined treatment with low-dose GCs can effectively induce and maintain MCD remission in MCD patients without acute kidney injury. Short-term adequate hormone-induced remission combined with RTX maintenance is also an effective therapy for MCD patients with acute kidney injury.

**Potential impact:**

- RTX, with or without GC, can be applied as an alternative to GC therapy for MCD patients.

## INTRODUCTION

Nephrotic syndrome (NS) encompasses a group of clinical syndromes induced by a variety of causes, mainly manifesting as massive proteinuria (>3.5 g/d), hypoproteinaemia (<30 g/l), high oedema, and hyperlipidaemia [1]. Among these, minimal change disease (MCD) is a common pathological type of NS that mainly occurs in children, accounting for ∼15% of adult NS incidence [2]. The pathogenesis of MCD remains unclear, but there is evidence to support that T-cell dysregulation leads to podocytopathy [3–6]. A randomized controlled trial by Boumediene *et al.* confirmed that MCD usually reoccurs after viral infection, immunization, or allergen exposure, suggesting that innate T cells, such as natural killer cells, may be involved in the pathogenesis [7]. The effectiveness of drugs used to treat B-cell depletion has also suggested a role for B cells in the pathogenesis of the disease [8]. Over a long period of time, total immunoglobulin G (IgG) and IgG subclasses change in NS patients with MCD. The activation promoter of B cells, plasma-soluble CD23, increases during the relapse of the disease. Evidence suggests that B cells are involved in the pathogenesis of NS (including MCD). Therefore, we conclude that both T and B cells are involved in the pathogenesis of MCD [5].

The 2021 Kidney Disease: Improving Global Outcomes guidelines recommended high-dose glucocorticoid (GC) therapy as the primary initial treatment for MCD in the absence of contraindications. However, such treatment frequently causes diabetes, lung infection, appearance change, acne, hypokalaemia, gastric ulcer, osteoporosis, and other adverse reactions. When corticosteroid contraindications are present, most patients can achieve clinical remission with cyclophosphamide, calcineurin inhibitors (CNIs), mycophenolate esters or mycophenolate sodium plus low-dose hormonal alternatives [9]. More than 80% of adult patients with MCD achieve remission during treatment with high-dose corticosteroids [10]. Patients with MCD usually relapse, and up to 33% may have frequent relapses or become corticosteroid dependent [11].

Rituximab (RTX) is a human/mouse chimeric monoclonal IgG1 antibody. It mainly targets the CD20 antigen on the surface of human B lymphocytes and specifically binds to it to deplete CD20-positive B cells. Originally approved for the treatment of non-Hodgkin's lymphoma, RTX has been increasingly applied in recent years to various autoimmune and kidney diseases [8, 12], such as membranous nephropathy and MCD. RTX can restore the normal immune system by indirectly regulating the T-cell network by affecting B cells [13], and it directly affects T cells and influences cellular immune homeostasis [14]. Furthermore, RTX has been shown to stabilize the actin cytoskeleton by directly binding to podocyte SMPDL3b to prevent its down-regulation *in vitro*, and its anti-proteinuria effect may be independent of B-cell depletion [15, 16]. There have been several studies on the use of RTX to reduce relapse in patients with frequent relapse or steroid-dependent disease. Research shows that RTX can significantly reduce relapse after treatment, with remission rates of 61%–100% at 1 year, and reduce the use of hormones and immunosuppression [9, 13, 17, 18]. RTX can be preferentially used in patients with a high risk of developing adverse effects to corticosteroids [19]. Evidence showing that RTX directly induces remission in MCD patients remains limited.

In the present study, domestically produced RTX injections by Shanghai Fosun Pharmaceutical Company (Shanghai, China) were used as the main observation drug. This anti-CD20 monoclonal antibody was tested for equivalence with imported RTX produced by Roche Diagnostics GmbH [20]. In view of the rare reports of the induction of MCD remission in adults by full-dose RTX alone or short-term combined treatment with GC, for the first time, we retrospectively analysed 21 MCD patients. The patients were assigned to an RTX induction therapy group, a short-term, low-dose GC combined with RTX induction therapy group or a short-term, full-dose GC combined with RTX maintenance therapy group. The remission and recurrence rates within 12 months were noted. The evidence of different treatment regimens inducing remission in MCD patients will provide new options and approaches for remission induction and the maintenance treatment of MCD.

## MATERIALS AND METHODS

### Subjects

A total of 26 adult patients with MCD admitted to the Department of Nephrology of Shandong Provincial Hospital from April 2020 to April 2022 were enrolled in this study. These patients had been diagnosed with MCD by renal biopsy and electron microscopic report and chose to be treated for MCD with sufficient RTX alone or short-term treatment combined with GC. After excluding two patients who did not receive standard RTX treatment and three who were followed up for <1 year, a total of 21 patients were included in the study. All patients had clinical manifestations of NS, with 24-hour urinary protein >3.5 g/24 h and serum albumin <30 g/l [2].

**Figure 1:**
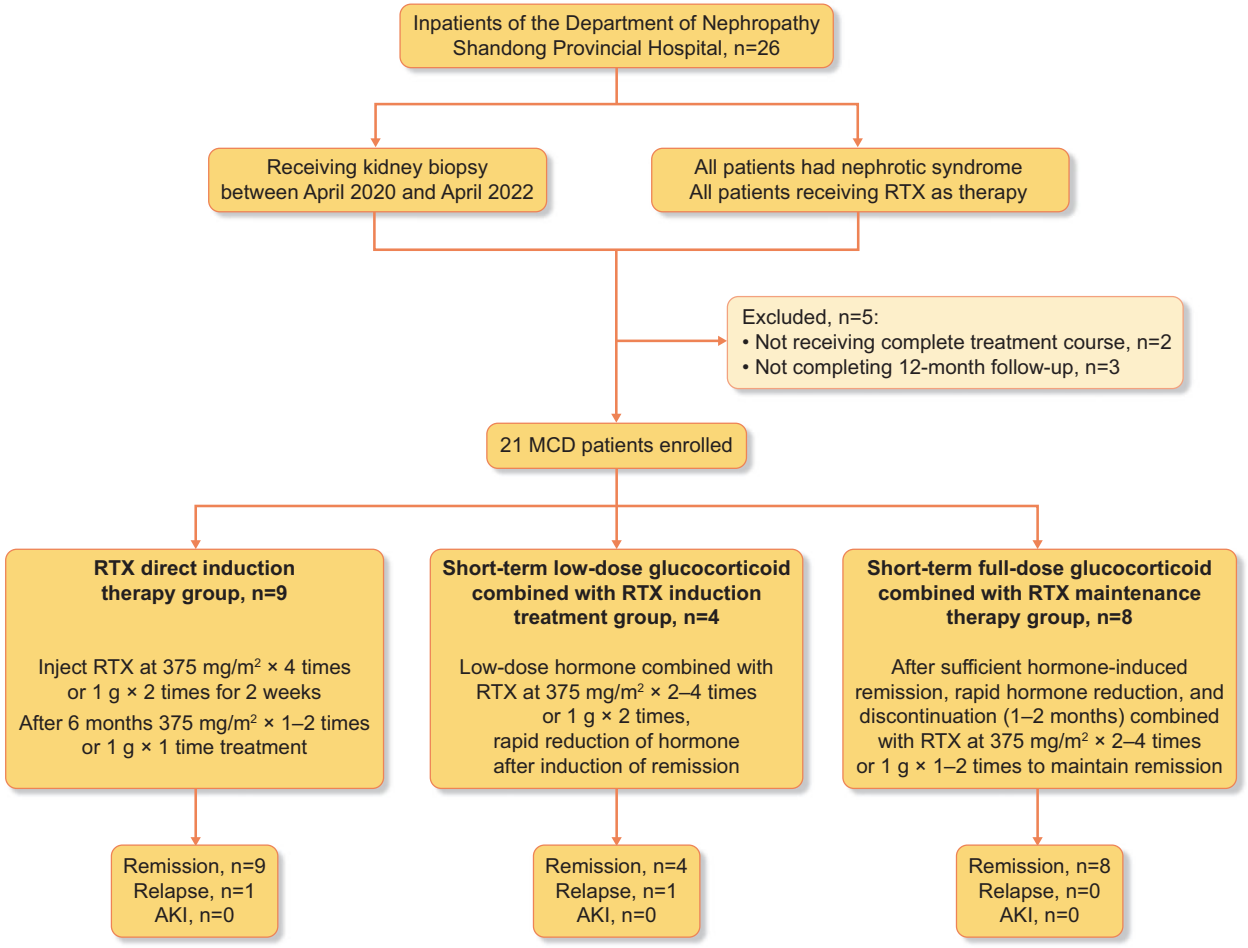
Flow chart of patients with MCD receiving rituximab (RTX) therapy. There were 21 MCD patients enrolled, of whom nine received RTX as direct induction therapy, four received low-dose hormone and RTX therapy, and eight received adequate doses of hormone and RTX to reduce recurrence. One patient relapsed in the direct induction therapy group, and another in the low-dose hormone and RTX therapy group. No patients exhibited AKI.

The patients were assigned to three groups according to different treatment regimens. The first group was the RTX direct induction therapy group (Group A), to which four patients were initially induced and in which four patients displayed a frequent relapse type and one exhibited a steroid-dependent relapse type. In this group, the eGFR was above 90 ml/min, the urine volume was above 800 ml and there was no acute kidney injury (AKI). The second group consisted of short-term, low-dose steroids combined with RTX induction therapy (Group B), and all patients displayed frequent relapse and were dependent on steroids or other immunosuppressants [cyclophosphamide (CTX) or CNI]. Before RTX treatment, two patients were in a complete relapse state and two was in a partial relapse state, while the eGFR in this group was also above 90 ml/min. The urine volume was above 800 ml, and there was no AKI. The third group was short-term, full-dose GC-induced remission combined with RTX maintenance therapy (Group C). All patients displayed frequent relapse, were dependent on steroids or other immunosuppressants (CTX or CNI) and were in complete relapse before receiving RTX treatment. All patients had severe proteinuria above 10 g/24 h, albumin below 20 g/l or AKI. Four patients had AKI. A total of nine patients were enrolled in Group A, four in Group B, and eight in Group C (Fig. 1). This study complied with the Declaration of Helsinki and was approved by the Ethics Committee of Shandong Provincial Hospital (SWYX: no. 2023–310). Written informed consent for participation was not required for this study, in accordance with national legislation and institutional requirements.

### Clinical data

Basic information included sex and age. Laboratory tests included urine protein, serum albumin, total cholesterol, serum creatinine, white blood cell, lymphocyte, CD19^+^ B cell, and CD20^+^ B cell counts as well as IgG, IgA, and IgM, among others. Follow-up data included follow-up time, remission status, relapse times, and adverse reactions.

### Treatment methods

There were two RTX administration regimens used in this study. The first included once-weekly 375 mg/m^2^ RTX intravenous injections for 4 weeks. The second consisted of two 1 g RTX intravenous injections with an interval of 2 weeks between them. These regimens have been shown to have the same effect on other diseases, with no statistical difference [12]. B-cell depletion was defined as a CD19^+^ lymphocyte count below 5 B cell/mm^3^. The patients in group A were treated with 4 × 375 mg/m^2^ RTX alone once weekly or with 2 × 1 g RTX over 2 weeks. After 6 months, these patients received 1–2 × 375 mg/m^2^ or 1 × 1 g of treatment. The patients in Group B were treated with short-term, low-dose GC (0.2–0.4 mg/kg) combined with either 4 × 375 mg/m^2^ RTX once weekly or with 2 × 1 g RTX over 2 weeks to induce remission, and the GC was subsequently rapidly reduced (within 4 months). The patients in Group C were treated with a full dose of GCs (1 mg/kg for 2–4 weeks). After the remission of proteinuria, the patients were given 2–4 × 375 mg/m^2^ RTX or 1–2 × 1 g RTX over 2 weeks. RTX was used as a saving agent for steroids. Following completion of the first induction therapy with RTX, the dosage of steroids was rapidly reduced and stopped (six tablets after completion of the first induction therapy with RTX, reduced by one tablets each week until withdrawal). The patients were followed up every 3 months, and the monitoring indicators included blood and urine routines, liver and kidney function, blood lipids and glucose, 24-hour urinary protein quantification, and circulating B cells. RTX-related adverse events were recorded during infusion and throughout the follow-up period. For the two administration schemes, it was decided according to the degree of B-cell recovery and clinical remission whether to give 375 mg/m^2^ or 1 × 1 g injection again at 6 and 12 months after treatment. The specific RTX medication method was the same as above, and patients were followed up at 2 weeks and 1, 2, 3, 6, 9, and 12 months after RTX treatment. Complications and recurrences were observed and recorded. The primary endpoints were the time to induced response and the clinical response rate at 12 months in each group, and the secondary endpoints were safety and the incidence of side effects.

Definition of indicators

(i) Time to achieve remission

The time from the first RTX injection to urine protein becoming negative.

(ii) Complete remission

Urine protein decreased to <0.3 g/d or urine protein/creatinine (PCR) <300 mg/g (or <30 mg/mmol); creatinine stabilized and serum albumin >3.5 g/dl (or 35 g/l).

(iii) Partial remission

Decrease in urinary protein to <0.3–3.5 g/d or urinary protein/creatinine <300–3500 mg/g (or <30–350 mg/mmol); and >50% decrease from baseline, or decrease in urinary protein to 0.3–3.5 g/d and serum albumin >30 g/l.

(iv) Relapse

Urine protein >3.5 g/d or urine protein/creatinine >3500 mg/g (or >350 mg/mmol) occurred after CR.

(v) Frequent recurrent type MCD

Total of ≥2 recurrences within 6 months (or ≥4 recurrences within 12 months).

(vi) corticosteroid dependent MCD

Recurrence during or within 2 weeks of completion of hormone therapy.

(vii) No remission

Heavy proteinuria (>3.5 g/day), a nonsignificant decrease in urinary protein (<50% decrease from baseline), and/or a significant increase in serum creatinine (>50% increase from baseline).

(viii) AKI

Elevation of creatinine to ≥0.3 mg/dl within 48 hours or ≥50% increase in creatinine from basal value within 7 days or decreased urine output [<0.5 ml/(kg/h)], lasting ≥6 hours].

(ix) Renal deterioration

Post-treatment rise in serum creatinine to >133 µmol/l or doubling of baseline serum creatinine level lasting >3 months.

(x) End-stage renal disease

Creatinine clearance <15 ml/min at last follow-up, initiation of dialysis, or renal transplantation.

(xi) Adverse reactions

The most common adverse reactions were infusion-related adverse reactions, including rash, erythema, pruritus, runny nose, and restlessness [21, 22].

(xii) Serious adverse events

Clinical mortality or life-threatening diseases such as severe pulmonary infection, pulmonary embolism, cerebral infarction, myocardial infarction, or hospitalization [21, 22].

### Statistical methods

SPSS v.22.0 software was used for the statistical analysis. The continuous variables with normal distribution are expressed as mean ± standard deviation (SD), and the *t*-test was applied for comparison between the two groups of data. The non-continuous variables that did not meet the normal distribution are expressed as median (interquartile range), and the rank sum test was used to compare the data between the two groups. A one-way analysis of variance was applied to compare multiple groups (≥3) of continuous variables between groups. Categorical variables are expressed as frequencies, and the *χ* test was used for comparison between groups. All test probabilities were two-tailed, and the significance level was set at 0.05. *P* < 0.05 was considered to indicate a significant difference, and *P* < 0.01 was considered to indicate a very significant difference.

## RESULTS

### Baseline data

A total of 21 adult patients with MCD (average age 34.67 ± 14.92 years) were included in this study. Before treatment, the average 24-hour urinary protein level of all patients was 11.36 ± 6.08 g/24 h, and the median serum albumin level was 19.50 (17.70, 27.85) g/l. The average serum creatinine level was 76.60 ± 22.19 µmol/l, and the eGFR was 102.64 ± 30.46 ml/min/1.73 m^2^. The CD19^+^ B lymphocyte count was 500.67 ± 275.34/µl (see Table 1 for details). Eleven patients (52.38%) received repeated RTX injections (375 mg/m^2^/l or 1 g/l) half a year after the completion of full-dose (4 × 375 mg/m^2^ or 2 × 1 g) RTX treatment.

**Table 1:** Baseline characteristics of patients with MCD included in the study.

Characteristic	Total (*n* = 21)	A (*n* = 9)	B (*n* = 4)	C (*n* = 8)	*P*
Male sex, *n* (%)	8(38.10)	3 (33.33)	2 (50.00)	3 (37.50)	0.868
Age (years)	34.67 ± 14.92	34.78 ± 16.45	31.50 ± 10.85	36.13 ± 16.39	0.890
Urine RBC/µl	2.60 (1.20, 6.71)	2.70 (1.39, 12.31)	2.03 (0.43, 11.16)	2.89 (0.88, 6.22)	0.581
Proteinuria (g/24 h)	11.36 ± 6.08	5.94 ± 1.98	5.19 ± 4.02	12.57 ± 5.59	0.304
WBC (×109/l)	7.45 (6.24, 10.12)	6.53 (5.37, 9.22)	8.15 (5.89, 12.71)	8.33 (7.19, 11.44)	0.909
Platelet (×109/l)	275.86 ± 72.10	297.56 ± 96.62	247.75 ± 44.88	265.50 ± 46.29	0.475
AST (µ/l)	20.50 (16.25, 30.00)	21.00 (17.50, 29.00)	15.50 (11.25, 22.00)	27.00 (19.00, 78.00)	0.297
ALT (µ/l)	22.00 (15.75, 38.75)	20.00 (14.50, 22.00)	32.00 (11.50, 60.00)	26.00 (23.00, 42.00)	0.420
Total protein (g/l)	47.94 ± 8.39	45.78 ± 7.29	56.40 ± 11.31	46.14 ± 5.89	0.073
Albumin (g/l)	19.50 (17.70, 27.85)	18.00 (17.20, 28.75)	30.80(20.30, 37.10)	19.25(17.27, 21.15)	0.052
Globulin (g/l)	24.03 ± 3.39	23.52 ± 3.39	25.08 ± 2.88	24.08 ± 3.88	0.766
BUN (mmol/l)	5.40 (4.80, 8.00)	5.20 (4.58, 5.40)	5.45 (4.90, 7.50)	6.60 (4.20, 9.30)	0.402
Serum creatinine(μmol/l)	76.60 ± 22.19	67.02 ± 17.29	76.88 ± 10.24	87.24 ± 27.80	0.176
eGFR (ml/min/1.73 m^2^)^a^	102.64 ± 30.46	111.74 ± 21.45	106.75 ± 25.22	91.49 ± 39.23	0.417
Cholesterol (mmol/l)	9.96 ± 3.43	10.13 ± 3.93	7.73 ± 2.13	11.46 ± 2.98	0.279
Lymphocyte count (×109/l)	2.40 ± 1.14	2.24 ± 0.94	3.01 ± 1.92	2.29 ± 0.94	0.525
Absolute values of CD19 (/µl)	500.67 ± 275.34	417.89 ± 263.94	639.42 ± 199.17	549.86 ± 333.73	0.470

Values are presented as number (%), median (interquartile range) or mean ± SD.

WBC, white blood cell; AST, aspartate aminotransferase; ALT, glutamic-pyruvic transaminase; BUN, urea nitrogen.

a The eGFR was calculated according to the Chronic Kidney Disease Epidemiology Collaboration equation.

The albumin level in Group A was lower than that in Groups B and C, with no statistically significant difference between the Groups (*P* = 0.06). Patients in Group C had higher proteinuria levels than those in Groups A and B, with no significant difference between the groups (*P* = 0.30).

### Analysis of the efficacy and follow-up data of 21 patients after treatment for MCD

**Figure 2:**
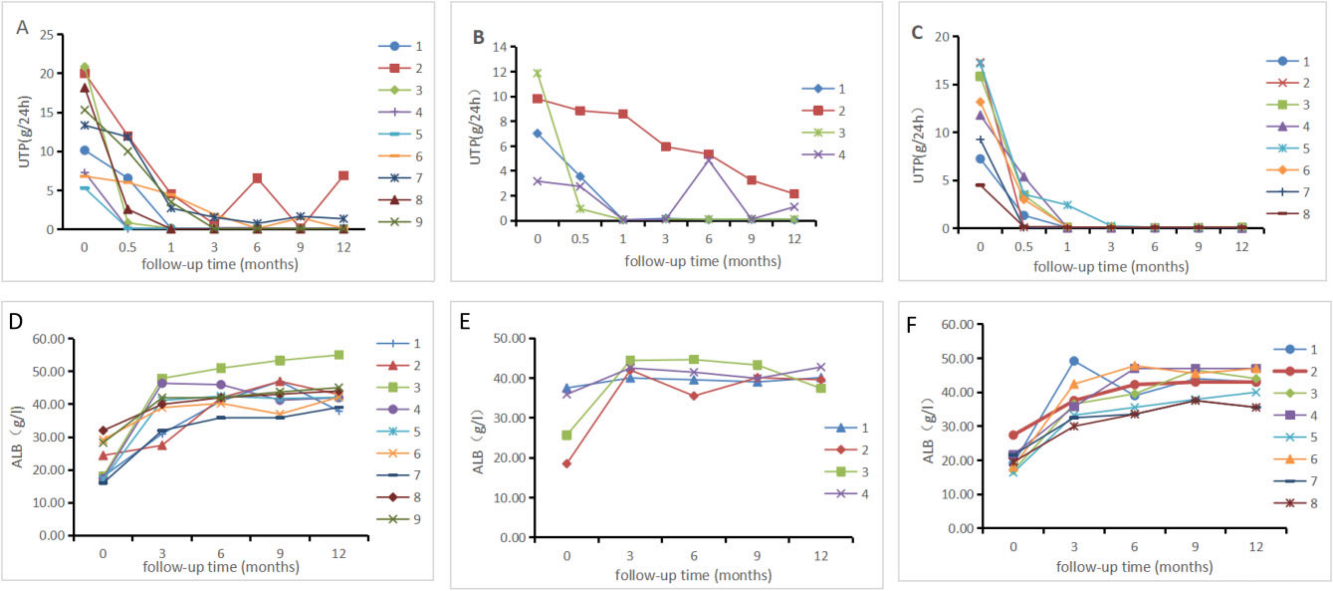
Data trend of albumin and 24-hour urinary protein quantity after rituximab treatment in patients who had been followed up for 12 months. UTP, 24-hour urinary protein quantity; ALB, albumin. There were 21 MCD patients enrolled, of whom nine were assigned to the RTX direct induction therapy group (**A**), four to the low-dose hormone and RTX therapy group (**B**), and eight to the adequate doses of hormone and RTX reduced recurrence group (**C**). The 21 patients exhibited an increasing trend in albumin levels and a decreasing trend in 24-hour urinary protein quantity levels.

A total of 21 adult MCD patients were followed up for 12 months. During the treatment period, all 21 patients (100.00%) achieved clinical remission, with 19 (90.48%) achieving CR and two (9.52%) achieving partial remission, with a median remission time of 4 (2.5, 12) weeks. Two patients relapsed within 1 year of follow-up, and the recurrence rate was 9.52%. In Group A, nine patients (100%) achieved clinical remission, of whom eight (88.89%) achieved clinical CR and one (11.11%) achieved partial remission, with a median remission time of 3 (2.25, 14) weeks, and one patient relapsed twice within the 1-year follow-up period. The relapsed patient relapsed once due to infection at 6 months after completion of full-dose RTX treatment alone and was relieved after treatment with 1 g RTX. In Group B, four patients (100.00%) achieved clinical remission, of whom three patients (75.00%) achieved CR, with a median remission time of 4 [4, 10] weeks, and one patient (25.00%) achieved partial remission. One patient continued to exhibit proteinuria after completing a full dose of 4 × 375 mg/m^2^ RTX treatment. This patient received a repeated 375 mg/m^2^ RTX injection once at a follow-up of ∼6 months after the completion of adequate RTX treatment, and the patient's proteinuria still did not resolve. In Group C, eight patients (100.00%) achieved clinical CR, and the median remission time was 3.5 [2, 4] weeks (Fig. 2). Three patients received additional doses of 375 mg/m^2^ RTX at 6 months after the completion of full-dose RTX treatment, and all patients discontinued steroids, with none relapsing. The details are presented in Table 2.

**Table 2:** Remission and relapse in patients.

Characteristic	Total (*n* = 21)	A (*n* = 9)	B (*n* = 4)	C (*n* = 8)
Time to achieve remission (weeks)^a^	4 (2.5, 12)	3 (2.25, 14)	4 (4, 10)	3.5 (2, 4)
Total remission, *n* (%)	21 (100.00)	9 (100.00)	4 (100.00)	8 (100.00)
CR, *n* (%)^b^	19 (90.48)	8 (88.89)	3 (75.00)	8 (100.00)
Partial remission, *n* (%)^c^	2 (9.52)	1 (11.11)	1 (25.00)	0 (0)
Relapse, *n* (%)^d^	2 (9.52)	1 (11.11)	1 (25.00)	0 (0)

Values are presented as number (%), median (interquartile range) or mean ± SD.

a Duration of remission: The time from the first RTX injection to urine protein becoming negative.

b Complete remission: A decrease in urine protein to <0.3 g/day or urine protein to creatinine <300 mg/g (or <30 mg/mmol); the serum creatinine was stable, and the serum albumin was >3.5 g/dl (or 35 g/l).

c Partial remission: Decrease in urinary protein to <0.3–3.5 g/d or urinary protein/creatinine <300–3500 mg/g (or <30–350 mg/mmol), and >50% decrease from baseline or decrease in urinary protein to 0.3–3.5 g/d and serum albumin >30 g/l.

d Relapse: Urinary protein >3.5 g/day or urinary protein/creatinine >3500 mg/g (or >350 mg/mmol) occurred after CR.

### Comparison of the laboratory indicators of RTX before and after treatment

Compared with the clinical data of adult MCD patients 1 year before RTX treatment, the amount of urinary protein in patients with RTX treatment for 1 year decreased significantly, the amount of albumin increased, lipid and protein metabolism disorders were significantly reduced, and the cumulative amount of hormones was significantly diminished. During the 12-month follow-up period, the 24-hour urinary protein quantification and CD19 absolute values of all MCD patients displayed a downward trend, while the serum albumin showed an upward trend. The results of the 12-month follow-up showed that the 24-hour urinary protein level of all MCD patients decreased from an average of 11.36 ± 6.08 g/24 h to 0.56 ± 1.54 g/24 h. Serum albumin gradually increased from 19.50 (17.70, 27.85) g/l to 42.20 (39.31, 44.03) g/l, and there were no statistically significant differences between the groups before or after treatment. The details are presented in Table 3.

**Table 3:** Final patient follow-up data.

Characteristic	A (*n* = 9)	B (*n* = 4)	C (*n* = 8)	*P*
Albumin (g/l)	42.20 (40.54, 44.57)	39.83(37.94, 42.08)	43.00 (36.66, 46.25)	0.434
Proteinuria (g/24 h)	0.97 ± 2.26	0.60 ± 1.03	0.07 ± 0.03	0.509
Absolute values of CD19 (/µl)	164.96 ± 153.39	67.24 ± 85.10	251.43 ± 202.86	0.245
Serum creatinine (μmol/l)	63.04 ± 11.10	58.60 ± 7.77	68.64 ± 9.53	0.333

Values are presented as number (%), median (interquartile range) or mean ± SD.

The albumin level in Group B was lower than that in Groups A and C, and there was no statistically significant difference between the groups (*P* = 0.07). Patients in Group B had higher proteinuria levels than those in Groups A and C, and there was no significant difference between the groups (*P* = 0.06).

### Safety

Adverse events occurred in six patients (28.57%) during the induction therapy of RTX with or without steroids and during follow-up; of these, one (4.76%) had a serious adverse event (interstitial pneumonia with hospitalization). The most common adverse events associated with RTX were infusion-related, including rash, erythema, pruritus, rhinorrhoea, and restlessness, which could be relieved by adjusting the infusion speed [21, 22]. After symptomatic treatment of hormone-related adverse reactions, all patients recovered. The details are presented in Table 4.

**Table 4:** Adverse events in all patients with MCD receiving rituximab.

Events	Total patients (*n*)	A	B	C
Any adverse event	6	1	0	5
Serious adverse events	1	0	0	1
Fatal	0	0	0	0
Non-fatal	6	1	0	5
Pulmonary infection	1	0	0	1
Non-serious adverse events	5	1	0	4
Infusion reactions*	1	1	0	0
Allergic eruption	1	1	0	0
Hormone-related adverse reactions	4	0	0	4
Weight gain	1	0	0	1
Dyslipidaemia, pathoglycaemia	1	0	0	1
Abnormal electrolyte, liver function	1	0	0	1
Purple striae	1	0	0	1
Alopecia	1	0	0	1

* Infusion reactions include bronchial wheezing, rash, erythema, itching, rhinorrhoea, and dysphoria.

**Table 5:** The number of patients of RTX given additionally after the initial induction dose and the doses of RTX.

Characteristic	Total (*n* = 21)	A (*n* = 9)	B (*n* = 4)	C (*n* = 8)
Total patients, *n* (%)	11 (52.38)	5 (55.56)	3 (75.00)	3 (37.50)
Number mid follow-up, *n* (%)	11 (52.38)	5 (55.56)	3 (75.00)	3 (37.50)
Number late follow-up, *n* (%)	3 (14.29)	3 (33.33)	0 (0)	0 (0)

a The follow-up of patients was decided according to the degree of B-cell recovery and clinical remission whether to give 375 mg/m^2^ or 1 × 1 g injection again at 6 and 12 months after treatment. One patient in Group A relapsed at 6 months of follow-up and was given 1 g of rituximab, while the remaining patients were given 0.5 g of rituximab.

## DISCUSSION

At present, in the treatment of MCD, RTX is primarily used in the area of relapse reduction in patients with MCD who are corticosteroid dependent or have frequent relapses over time. In these patients, RTX treatment with minimal hormone and/or immunosuppressant maintenance can help most patients successfully reduce and stop GC and immunosuppressant use, and can also reduce the number of patients with recurrence [9, 13, 17, 18]. The results of this study showed that the induction regimen of full-dose RTX alone or combined with low-dose steroids can achieve clinical remission in MCD patients without AKI, avoid the side effects of steroids, and significantly reduce the recurrence in patients. For MCD patients with AKI, we reported for the first time that the treatment regimen of early administration of sufficient GC to induce remission, adding RTX after proteinuria remission, and then rapidly reducing and stopping GC could significantly reduce the dosage of GC and the relapse of MCD.

This study showed that 100.00% of 21 adult MCD patients achieved clinical remission at the end of the follow-up period. Among the 13 MCD patients without AKI (RTX alone or in combination with small doses of hormones), 84.62% achieved CR and 15.38% achieved partial remission. This is consistent with the report of Fenoglio [19]. The present study also had more success than the 77.77% remission rate of nine adult-onset MCD patients reported by Guan *et al.* [23], and the reason for the low remission rate in this study may be associated with the significantly lower RTX dose used by the patients. Compared with conventional steroid-applied MCD treatment, the overall remission rate of MCD induced by full-dose RTX alone or in combination with low-dose steroids was superior to the 74.85% remission rate of 95 patients reported by Waldman *et al.* [24], and consistent with the remission rate of 23/25 patients (92.00%) reported by Medjeral-Thomas *et al.* [25]. Therefore, the findings of the present study suggest that full-dose RTX alone or in combination with low-dose GCs induces remission of MCD and may be a new induction regimen for patients with MCD.

The median remission time of the 21 adult MCD patients was 4 weeks, consistent with the 3.5 weeks reported by Guan *et al*. who treated MCD with RTX alone [23]. Consistent with a median time to CR of 3–4 weeks in the prednisone group [24, 25], the present study further demonstrated that RTX and GC regimens are similar in terms of the time to remission. In our cohort of 17 patients with corticosteroid dependent and frequent relapses—a number lower than that of patients who relapsed again in previous corticosteroid dependent patient studies—only 9.52% of patients experienced MCD relapse in our 1-year follow-up cohort, which was lower than in the use of RTX to reduce relapse reported in many recent studies [26]. Almost all frequently relapsing or corticosteroid dependent patients treated with RTX experience relapse after recovery from RTX-induced peripheral B-cell depletion [27]. However, RTX injections help to maintain remission [28]. A study of patients with frequent relapses and corticosteroid dependent MCD (recurrence during or within 2 weeks of completion of hormone therapy) showed a significant reduction in relapse rates after B-cell-count recovery with 1–2 additional doses of RTX therapy compared with pretreatment [29]. The present study had a lower recurrence rate, the reason for which may be related to our previous application of sufficient RTX and 6 months of supplementary RTX treatment to provide good control over recurrence.

RTX was well tolerated by most patients in this study, and only one patient had a serious adverse event. The side effects of RTX were significantly lower than those reported in the prednisone group and the steroid combined with tacrolimus group in previous studies [25]. Other studies have reported that the incidence of serious adverse events can be between 0% and 17%, among which the most common adverse reactions were infusion reactions, which can be alleviated by adjusting the infusion speed. In the present study, infusion reactions were relatively rare, which may be due to strengthening the pretreatment and limiting the infusion speed to avoid infusion-related events or improve them to a certain extent [21, 22]. In many studies, RTX has been well tolerated, with the serious side effects being rash, drug eruption, infusion reaction, leukopaenia, and pneumonia. The incidence of serious adverse events was 0.092 per year, and the RTX measurement was positively correlated with the incidence of adverse events [30]. All the adverse reactions in the cases included in the present study were non-fatal, and this study fully confirmed the safety of RTX in the treatment of MCD in adults.

This study had several limitations. First, this was a single-centre retrospective study, and the sample size was too small to answer all the questions. Second, the induction protocol also had certain drawbacks. Despite these limitations, our study remains of interest, providing new ideas and approaches for resolving initial and recurrent MCD patients, and also provides new means for maintaining remission of MCD. Overall, this study provides important data support for future large-scale prospective studies.

In conclusion, full-dose RTX alone or short-term RTX combined with low-dose GC can effectively induce and maintain remission of MCD and can be applied as an alternative to GC therapy. Short-term adequate GC-induced remission combined with RTX maintenance therapy is also an effective alternative. Different induction regimens for MCD patients with different backgrounds need further investigation.

## Data Availability

The data supporting the findings of this study are openly available in figshare at DOI:10.6084/m9.figshare.24162750.
